# Being overburdened and medically underserved: assessment of this double disparity for populations in the state of Maryland

**DOI:** 10.1186/1476-069X-13-26

**Published:** 2014-04-04

**Authors:** Sacoby Wilson, Hongmei Zhang, Chengsheng Jiang, Kristen Burwell, Rebecca Rehr, Rianna Murray, Laura Dalemarre, Charles Naney

**Affiliations:** 1 Community Engagement, Environmental Justice and Health, University of Maryland-College Park, College Park, MD 20742, USA; 2 Maryland Institute for Applied Environmental Health, University of Maryland-College Park, College Park, MD 20742, USA; 3 Epidemiology, Biostatistics, and Environmental Health Science, School of Public Health, University of Memphis, Memphis, TN 38111, USA

## Abstract

**Background:**

Environmental justice research has shown that many communities of color and low-income persons are differentially burdened by noxious land uses including Toxic Release Inventory (TRI) facilities. However, limited work has been performed to assess how these populations tend to be both overburdened and medically underserved. We explored this “double disparity” for the first time in Maryland.

**Methods:**

We assessed spatial disparities in the distribution of TRI facilities in Maryland across varying levels of sociodemographic composition using 2010 US Census Health Professional Shortage Area (HPSA) data. Univariate and multivariate regression in addition to geographic information systems (GIS) were used to examine relationships between sociodemographic measures and location of TRI facilities. Buffer analysis was also used to assess spatial disparities. Four buffer categories included: 1) census tracts hosting one or more TRI facilities; 2) tracts located more than 0 and up to 0.5 km from the closest TRI facility; 3) tracts located more than 0.5 km and up to 1 km from a TRI facility; and 4) tracts located more than 1 km and up to 5 km from a TRI facility.

**Results:**

We found that tracts with higher proportions of non-white residents and people living in poverty were more likely to be closer to TRI facilities. A significant increase in income was observed with an increase in distance between a census tract and the closest TRI facility. In general, percent non-white was higher in HPSA tracts that host at least one TRI facility than in non-HPSA tracts that host at least one TRI facility. Additionally, percent poverty, unemployment, less than high school education, and homes built pre-1950 were higher in HPSA tracts hosting TRI facilities than in non-HPSA tracts hosting TRI facilities.

**Conclusions:**

We found that people of color and low-income groups are differentially burdened by TRI facilities in Maryland. We also found that both low-income groups and persons without a high school education are both overburdened and medically underserved. The results of this study provide insight into how state agencies can better address the double disparity of disproportionate environmental hazards and limited access to health care resources facing vulnerable communities in Maryland.

## Introduction

Environmental injustice is driven by privilege, power-- particularly structural and environmental racism which are embedded in our regulatory schema, zoning, planning and community development processes [1,2]. Additionally, unhealthy geographies that concentrate environmental, social, and health risks in urban and rural areas are produced and are known as ‘riskscapes’ [3,4]. The original Toxic Waste and Race in America report published in 1987, was the first report to demonstrate that many economically underserved populations and people of color communities are disproportionately impacted by locally unwanted land uses (LULUs) [5]. Since the 1987 report and the recent twenty-year anniversary report [5], researchers have shown that these disparities persist, with low-income persons and populations of color continuing to live in communities with a differential burden of LULUs including toxic release inventory (TRI) facilities [6-18], landfills [5], incinerators [5], hazardous waste sites [5,18], sewer and water infrastructure including sewer and water treatment plants [7,8,19,20], coal-fired plants [5], industrial animal operations [21,22], and Superfund sites [23-25]. This disproportionate burden can lead to increased exposure to harmful environmental conditions and chemical, physical, and biological agents for impacted communities [1,2,26-28].

Previous research has also shown that populations of color and low-income groups living in poor environmental conditions have health risks due in part to various social determinants of health including segregation, racism, socioeconomic status (SES), income inequality, and inequities in planning and zoning [1-4,6,14,19,20,28-31]. Studies have shown that underlying social and economic vulnerabilities contribute to increased health disparities [29,31,32], which further enhance the long-term effects of environmental injustice. Environmental justice communities are also affected by a higher concentration of psychosocial stress [1,28,29] that can lead to an increase in community-level and individual-level stress.

A potential environmental justice issue in the state of Maryland is the distribution and concentration of TRI facilities. Previous studies in New York [7], South Carolina [9,10], Oregon [11,12], California [13,18], and the entire United States [6,8,15] have demonstrated the disproportionate burden of TRI facilities in low-income and non-white communities (often using census tracts as the unit of analysis). Ringquist found that TRI facilities were found in zip codes with large populations of people of color [15]. Racial composition of neighborhood was found to be a stronger contributor to the trend associated with the distribution of environmental risk than class [15]. Abel found that people of color and low-income residents were disproportionately closer to TRI facilities in metropolitan St. Louis [16]. Spatial concentration of residents of color averaged nearly 40% within one km of St. Louis TRI sites compared to 25% in other locations [16]. Fricker and Hengarter report that the racial/ethnic composition of a census tract in Metropolitan New York was positively associated with the presence of LULUs including TRI sites [7]. In metropolitan New York, the Hispanic population resided in neighborhoods closer to undesirable sites than other racial/ethnic groups [7]. Using 1990 US Census and 1990 TRI data, Daniels and Friedman observed a positive relationship between proportion Black residents and toxic releases to air [17].

Wilson et al. found significant burden disparities [9], where more TRI facilities were located in census tracts with higher non-white and low-income populations for the state of South Carolina and Metropolitan Charleston. In addition to this work, other researchers have documented similar racial and income disparities among communities hosting TRI facilities [11,12]. Neumann et al discovered that TRI facilities were located disproportionately in people of color neighborhoods and in areas with lower incomes compared to those in the surrounding counties [11].

Miranda et al. took these analyses one step further to scrutinize the effects of new TRI reporting requirements implemented in December 2006 which reduced reporting requirements for certain chemicals released in limited quantities [33]. Specifically, the study found that facilities given permissions to use a short reporting form were disproportionately located in majority non-white census tracts [33]. As a result, residents were losing access to salient information regarding chemical releases compared to their white counterparts who were mostly located in areas with more stringent reporting requirements.

The paucity of information on chemical releases is problematic because many of the chemicals typically emitted by TRI facilities and other LULUs have been linked to adverse cancer and non-cancer health effects [34,35] which may significantly impact people who live near these environmental hazards. For example, emissions from TRI facilities may include harmful substances such as benzene, cadmium, toluene, and mercury, among other chemicals [36,37]. Moreover, populations exposed to TRI-related chemicals may have an increased risk of adverse health outcomes such as low birth weight, asthma, and cancer [13,38-40] estimated by previous research using risk assessment methodology.

In addition to being differentially burdened by environmental hazards and LULUs, the lack of access to salutogenic infrastructure (e.g., positive and health-promoting features of the built and social environment) [1,2,41] is a major environmental justice issue for people of color communities and economically disadvantaged populations. Many persons of color live in socially disadvantaged areas with limited access to primary care resources [42-46]. Access to adequate healthcare may be a major problem for economically disadvantaged in communities with pre-existing burden, exposure, and environmental health disparities. A growing body of literature has examined the distribution of health enriching resources and medical care services across varying racial/ethnic and SES composition at the neighborhood level. Limited access to hospitals and medical professionals and lower quality of care both play major roles in health outcomes and disparities in disadvantaged neighborhoods [47-52]. Being both disadvantaged and medically underserved means disadvantaged populations may have higher rates of chronic conditions, more drug use, emotional problems, and worse health behaviors than other populations [53], but lack of access to competent high quality care may mean lower immunizations rates for children [54] and more hospitalizations for treatable and preventable conditions [55]. Taken as a whole, the differential burden of pollutogens and access to salutogenic resources has important implications for health and environmental health disparities [1,2,41].

The issue of how people of color neighborhoods and low-income populations are disproportionately burdened by LULUs such as TRI facilities and are potentially underserved due to poor access to medical infrastructure is important for a state like Maryland with a number of racial/ethnic and SES-related health disparities. The 2010 MD Plan to Eliminate Minority Health Disparities (MPEMHD) lists racial/ethnic disparities in healthcare utilization, access to primary care, and the burden of all-cause mortality, heart disease, renal disease, hypertension, obesity, HIV/AIDS, and asthma as critical areas for improvement [56]. Three of the four counties in MD with the highest population of persons of color (Baltimore City, Charles County, Montgomery County, and Prince George’s County) all have more than 50% non-white residents and the highest total environmental releases (Charles County, Prince George’s, and Baltimore City) [56]. As further proof that these health disparities are a serious problem, the Maryland Health Improvement and Health Disparities Reduction Act (MHIHDRA) was recently passed to address the aforementioned disparities [56].

In addition, the MD Department of Health and Mental Hygiene (MDHMH) uses its Environmental Public Health Tracking Network (EPHTN) to offer web-based data sharing tools for residents, policymakers, and other public officials to create their own maps and charts documenting environmental health disparities in their service areas [57,58]. Information available for analysis through the MD EPHTN includes the following: 1) childhood blood level testing, 2) myocardial infarction and asthma-related hospitalization data, and 3) low birth weight from state birth certificate records [57]. Thus, running a query reveals significant disparities in blood lead levels among counties; with Baltimore City having a much higher rate than other counties (449 1-year olds in 2008 had elevated blood lead levels versus many other counties that had none) [59]. Another query revealed that asthma hospitalization discharge rates were higher among blacks compared to whites across the state (36.79 per 10,000 vs. 11.20 per 10,000, respectively), and this disparity was more pronounced in certain areas (32.33 per 10,000 vs. 2.88 per 10,000 in Baltimore City and 10.31 per 10,000 vs. 1.00 per 10,000) [56,59].

The purpose of this study was to assess whether TRI facilities in Maryland were more likely to be located in census tracts with higher proportions of black, non-white, low-income, or less educated persons. In addition, we assessed whether populations near TRI facilities had limited access to health care infrastructure as indicated by health professional shortage area (HPSA) designation at the census tract level. By assessing both presence of TRI facilities and HPSA designation, we assessed the potential “double disparity” of being environmentally overburdened and underserved in terms of health care access across neighborhoods with varying sociodemographic composition.

## Methods and materials

### Study area

The state of Maryland (MD) is ranked 42^nd^ in size among states in the U.S., but 19^th^ in population, which makes it one of the more densely populated states in the nation [60]. The population within MD is concentrated in two main areas: 1) around the harbor in Baltimore County and Baltimore City and 2) Montgomery County and Prince George’s County, MD near Washington, DC. According to the 2010 US Census, there were 5,773,552 people living in MD with 61.1% white and 30.0% black [60]. Furthermore, populations of color living in MD are highly concentrated in these two areas. Baltimore City is 72% non-white and Prince George’s County is 85% non-white, while MD is 39% non-white as a whole [60].

The number of people living in poverty is also unevenly distributed throughout the state. For example, Maryland ranks 3^rd^ in the nation in median household (HH) income with only 8.6% of the state living in poverty (compared to 13.8% nationwide) [60]. In Baltimore City, the most densely populated urban area in the state, 21.3% of residents live below the federal poverty line [60]. In two other distinct parts of the state, Allegany County in western MD has 14.5% of its residents living in poverty and Dorchester County on the eastern shore has 13.4%, both well above the state poverty rate [60].

### Sociodemographic (SOD) Measures

This study used key demographics for MD modeled as quartiles from 2010 census data [9,61,62]. While SOD information is available at various geographic scales (ZIP code tabulation areas (ZCTAs), tracts, block groups, and blocks), we utilized census data at the tract level to enumerate the following population characteristics: race/ethnicity (% non-white includes all other races including Hispanics except non-Hispanic white; and % Hispanic) and variables related to socioeconomic status (SES). SOD measures included in our study were poverty (% population below poverty line), education (% population age greater than 25 years with < high school (HS) education), unemployment (% of population 16 years and older who were unemployed), homeownership (% of homes occupied by owners), and homes built before 1950 (% of homes built pre-1950). The variable related to income was median HH income. Median HH income, % poverty, house construction year, % unemployment and educational attainment (i.e., % without a HS diploma) were calculated using the 2006 to 2010 American Community Survey (ACS) 5-year estimates.

### USA today diversity index

The USA Today Diversity equation measures the distribution of multiple races. Specifically, it calculates the probability that any two people randomly selected in an area are from different races or ethnic groups [63]:

$$\mathrm{USA}\;\mathrm{Today}\;\mathrm{Diversity}=1-\left({\left(1-q_{h}\right)}^{2}+q_{h}^{2}\right)\times \sum p_{1}^{2}$$

where q_h_ is the % of Hispanics in an area and p_i_ includes % White, African-American, Asian, American Indian and Alaska Native, and Native Hawaiian and other Pacific Islander. The higher the Diversity Index, the more diverse a population in a particular region ranged from 0 to 100. The Diversity Index is well-suited for characterizing racial and ethnic diversity at the tract level, while other indices are more appropriate for examining segregation at larger geographic scales, such as the MSA. In this study, the Diversity Index was calculated at the census tract level.

### Toxics release inventory (TRI)

The TRI database was established by Section 313 of the 1986 Emergency Planning and Community Right-to-Know Act (EPCRA) [64]. Estimations of the mass of disposal or other release of over 650 chemicals were reported to the TRI by each facility (2010 Toxics Release Inventory national analysis overview). The 2010 single Facility Registry System (FRS) state files were downloaded from the USEPA and TRI facilities were selected from the FRS which contains their respective latitude and longitude coordinates. Distance between a TRI facility and nearest census tract was calculated in ArcGIS 10 (esri, Redlands, CA). A TRI facility located in a census tract or on the boundary of the census tract was assigned a distance of 0 which means that the census tract ‘hosts’ the TRI facility.

### Healthcare infrastructure

The Department of Health and Human Services (DHHS) created Health Professional Shortage Area (HPSA) designation to identify areas facing a critical shortage of providers [42-46,65]. A HPSA can be a distinct geographic area (such as a county), a specific population group within an area (such as low-income individuals), or a specific health care facility [66]. We obtained 2010 HPSA data for the state of Maryland and categorized each census tract in Maryland either as a HPSA census tract or non-HPSA census tract.

### Statistical and geographic methods

To assess the proximity from TRI facilities to different sociodemographic features, we used two approaches. In the first approach, census tracts were grouped by their distance to the nearest TRI facility. A distance was measured along a straight-line path between a facility and the point closest to the facility on the boundary of a census tract. Based on this definition, we grouped census tracts into four groups (or four distance bands), defined as: band 1 composed of census tracts hosting one or more TRI facilities (at least one TRI in the census tract and the distance was 0), band 2 included census tracts whose distance to the closest TRI facility was greater than 0 and up to 0.5 km, band 3 greater than 0.5 km and up to 1 km, and finally band 4 greater than 1 km and up to 5 km. Census tracts whose distance to the nearest TRI facility was greater than 5 km but within 10 km were excluded from this analysis due to the small number of census tracts in this distance band. Then within each band, the mean percentage of each SOD measure for those census tracts was calculated. This task was performed to evaluate how SOD composition changed with the change in distance. When disproportionality did not exist with respect to a specific SOD group, we expected that the mean SOD measure (by percentage) in each distance band would correspond to the mean percentage calculated for the entire state. Student t-tests were used to test this hypothesis of equality.

In the second approach, census tracts were grouped based on the percent number for a specific SOD variable. This was done to evaluate how distance changed with respect to the change in the composition of population for a specific SOD group. To this end, we divided the census tracts into four groups (Q1 to Q4) using quartiles of a SOD variable measured by percentage across the state of Maryland. Then in each group of census tracts, the average distance between each tract and the closest TRI facility was calculated.

To quantify the relationship between the distribution of TRI facilities and SOD factors at the census tract level, we first applied univariate linear regression to test the association of distance from census tracts to the closest TRI facility (dependent variable) with each individual SOD factor (independent variable). This was then followed by a multivariable linear regression model with all SOD factors included in order to each SOD factor’s effect after adjusting for other SOD factors, i.e., y_i_ = β_0_+ ***β***^T^***X*** + ϵ_i_ where y_i_ denotes distance, ***X*** denotes a vector of SOD factors, the ***β*** vector is for the SOD factor effects, and ϵ is the random error. To eliminate redundant variables, a stepwise variable selection approach based on the Bayesian Information Criterion (BIC) was applied to finally select the most important variables.

We hypothesized that low-income and people of color communities are both overburdened by environmental hazards and unhealthy land uses and underserved by health infrastructure known as the “double disparity”. To test the difference in SOD composition between HPSA tracts and non-HPSA tracts, we compared the average SOD measures in HPSA tracts and non-HPSA tracts in two distance band areas, one area covered census tracts hosting a TRI facility and the other area including census tracts within 1 km to 5 km to the nearest TRI facility. For each area, a student t-test was used to test the difference in composition for each SOD measure between HPSA tracts and non-HPSA tracts.

All of the above calculations and hypothesis testing were performed using R version 2.15.0 [11,12,67]. Statistical significance level was set at 0.05. TRI facilities were mapped and overlaid by select SOD features (% non-white and % poverty) in ArcGIS 10 (esri, Redlands, CA). Choropleth maps were created to illustrate the spatial relationship between TRI facilities and sociodemographic composition using quartiles.

## Results

**Figure 1:**
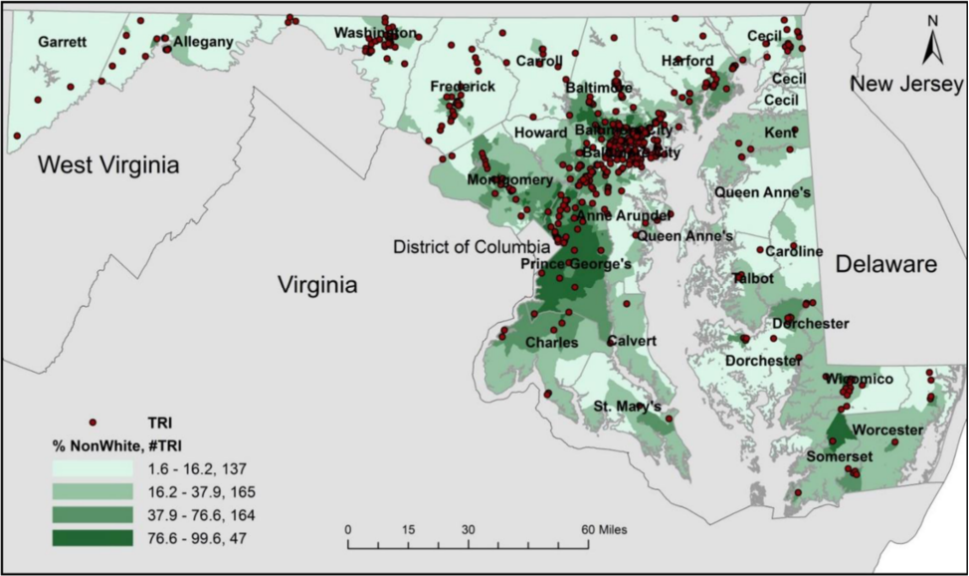
Choropleth Map of TRI Facilities in Maryland by Quartiles for Percent Non-White (2010 US Census).

**Figure 2:**
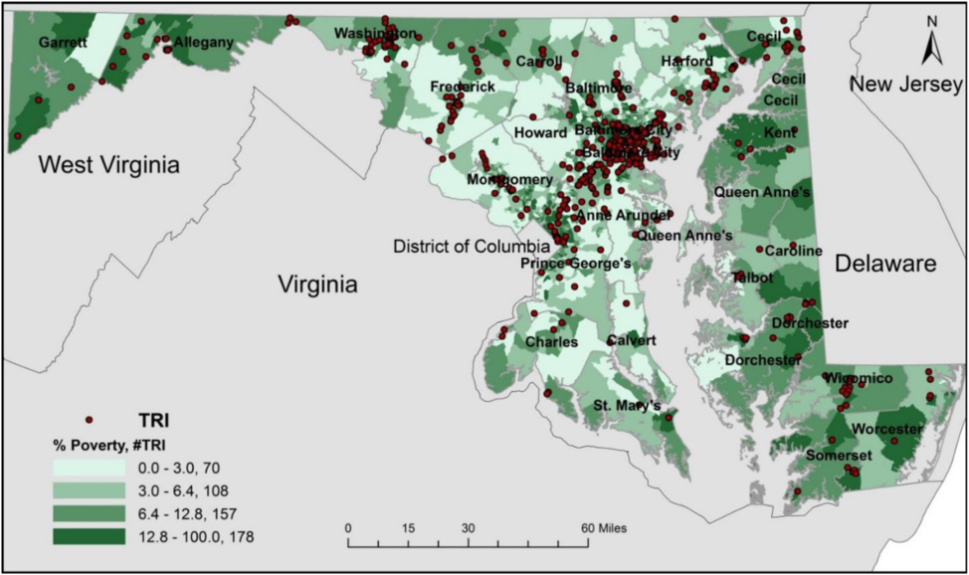
Choropleth map of TRI facilities in Maryland by quartiles for percent poverty (2010 US census).

Figures 1 and 2 were created to show the spatial distribution of TRI facilities in relation to composition of various sociodemographic groups at the census tract level. There were 525 TRI facilities located in 259 census tracts in MD out of a total of 1390 census tracts. In addition, there was one census tract located close to the Port in South Baltimore that hosts 14 TRI facilities which was the largest number of TRI facilities found in one tract in the state. The choropleth maps show clusters of TRI facilities in the Baltimore Metropolitan Statistical Area (MSA), Washington County (western MD), and Wicomico County (Eastern Shore).

For the purpose of this analysis, we only present mapping results for % non-white and % poverty due to the fact that previous studies have shown a positive relationship between number of TRI facilities and presence of non-whites and low-income persons. For % non‒white, 137 TRI facilities were located in the first quartile followed by 165, 164, and 47 facilities in the second, third, and fourth quartiles, respectively. There were 12 TRI facilities not included on the maps due to a locational error or they were located in census tracts where no people lived. The census tracts in the fourth quartile had the largest non-white population but the fewest TRI facilities compared to other quartiles. Regarding % living in poverty, 70 TRI facilities were located in the first quartile followed by 108, 157, and 178 facilities in the second, third, and fourth quartiles, respectively. Both figures show evidence of differences in the concentration of TRI facilities across varying levels of sociodemographic composition at the census tract level. Figure 2 indicated a clear linear relationship between % living in poverty and TRI facilities. Thus, as % persons in poverty increased, the number of TRI facilities increased.

Statistical summaries and tests indicated that the population distribution for different SOD variables changed with an increase in distance to TRI facilities (Table 1). For example, mean % non-white in census tracts having at least one TRI facility in their geographic boundary (host) (38.4%) was significantly lower than the mean % non-white in all census tracts in Maryland. With an increase in distance to the nearest TRI facility, % non-white increased and becomes statistically significantly higher than the statewide mean for % non-white. The census tracts with distance to the nearest TRI facility in the third distance band (>0.5 km and <1 km) had the highest % non-white (54.8%) which was 8.7% higher than the statewide average Results indicate that on average, the highest proportion of non-white residents was in tracts that were between 0.5-1 km distance from a TRI facility. However, there was no statistically significant difference between statewide mean % Hispanic and mean % Hispanic in host tracts or other tracts grouped by distance.

**Table 1:** Mean distribution of sociodemographic measures by TRI facility buffer zones in Maryland (2010 census)

**Sociodemographic measure**	**State**	**Host (a census tract that hosts at least one TRI facility)**	**0-0.5 km**	**0.5-1.0 km**	**1-5.0 km**
**% Hispanic**	7.8	6.9	8.5	7.8	8.6
**% Non-white**	46.1	38.4**	49.2	54.8**	49.1
**% Poverty**	9.6	11*	10.8	12.7**	8.3*
**% Unemployment**	7.1	7.2	8**	8.3**	6.6*
**% < HS education**	13.2	15.3**	14.6	16**	11.7**
**% Homeownership**	67.1	65.3	63.3*	59.3**	68.9
**% Homes built pre-1950**	20.8	25.4**	24.6*	28.7**	16.9**
^**a**^**Diversity index**	0.43	0.43	0.46*	0.43	0.44
**Median HH income**	74810	65239**	69399*	64362**	81190**
**MD census tracts (N)**	1390	259	218	192	613

*p-value <0.05 **p-value <0.01.

^a^The Diversity index was 0.428 for state and 0.433 for 0.5 to 1.0 km group.

Percent living in poverty in host tracts and % poverty of census tracts in the second distance band was almost the same (11% and 10.8%, respectively). They both were higher than the statewide average (9.6%). Percent living in poverty increased to 12.7% in the third distance band (>0.5 km and <1 km) and decreased to 8.3% for the fourth distance band (>1 km and <5 km). In addition, these results reveal that there were more persons in poverty living in areas closer to TRI facilities especially in census tracts within the third distance band (>0.5 km and <1 km). We observed a similar pattern for changes in % less than HS education as distance from census tracts to the nearest TRI facility increased (Table 1). For % unemployment, there were no statistically significant differences between statewide average percentage and in areas hosting TRI facilities (7.1% for statewide average and 7.2% for host tracts).

For housing-related variables, we observed lower % homeownership in host tracts compared to the statewide average, but the difference was not statistically significant. As the distance from census tracts to the nearest TRI facility increased, mean % homeownership decreased from 63.3% to 59.3% from the second distance band (>0 km and <0.5 km) to the third distance band (>0.5 km and <1 km) both of which were statistically lower than the statewide average (67.1%). After 1 km, % homeownership increased to 68.9% which was 1.8% higher than the statewide average. The changes in % homes built pre-1950 were similar to changes in % poverty (Table 1).

As for the Diversity index, no clear pattern was observed. Statistically significant differences were observed in the 0-0.5 km buffer (0.46). At the census tract level, the statewide average median HH income ($74,810) was almost $10,000 higher than median HH income for census tracts hosting a TRI facility and in areas in the second distance band (>0.5 km and <1 km). The median HH income increased to $81,190 in areas in the fourth distance band (>1 km and <5 km). These results indicate that TRI facilities possibly cluster in low-income areas that host the LULU or in areas located at least 1 km away from the nearest TRI facility.

Table 2 shows the mean distance between TRI facilities and each group of census tracts defined by quartiles of SOD measures. When considering race, the mean distance from TRI facilities to census tracts grouped by % Hispanic from Q1 to Q4 decreased 20%, while % non-white from Q1 to Q4 decreased 40% (2.5 km vs 1.5 km). These results indicate that census tracts with a higher % non-white population were located closer to TRI facilities. For % poverty, the mean distance from census tracts to the nearest TRI facility decreased 50% from Q1 to Q4. A similar pattern in distance change across different quartile groups of census tracts was observed for % unemployment and % less than HS education (Table 2). As for % homeownership, the higher the percentage, the farther the distance to the nearest TRI facility (mean distance of 1.2 km for Q1 and 2.7 km for Q4). Percent homes built pre-1950 showed a non-linear pattern. The distance first increased from Q1 to Q2, and then decreased from Q3 to Q4. The Diversity index showed a decreasing pattern of distance from Q1 to Q4, consistent with the pattern revealed by % poverty, % unemployment, and % less than HS education. The mean distance to the nearest TRI facility for census tracts grouped by quartiles of median HH income showed a linear increase from Q1 to Q3 with a rate of 0.4 km. The mean distance then increased to 2.6 km in the Q4 group which was almost 2.4 times farther away than the Q1 census tract group.

**Table 2:** Mean distance to TRI facilities by quartiles for various sociodemographic measures in Maryland (2010 census)

**Sociodemographic measures**	**Q1 (km)**	**Q2 (km)**	**Q3 (km)**	**Q4 (km)**	**Q4/Q1**
**% Hispanic**	2.1	1.9	1.6	1.6	0.8
**% Non-white**	2.5	1.7	1.4	1.5	0.6
**% Poverty**	2.3	1.9	1.8	1.1	0.5
**% Unemployment**	2.2	1.9	1.8	1.3	0.6
**% < HS education**	2.3	2.2	1.7	1	0.4
**% Homeownership**	1.2	1.3	2	2.7	2.3
**% Homes built pre-1950**	2	2.2	1.8	1.1	0.6
**Diversity index**	2.2	2	1.4	1.4	0.6
**Median HH income**	1.1	1.5	1.9	2.6	2.4

In the univariate regression model, all SOD measures were statistically significantly associated with distance to TRI facilities (Table 3). Across the state of MD, census tracts with a higher percentage of non-white residents demonstrated resistance to decaying distance between adjacent TRI facilities (Beta coefficient = -0.011; p < 0.001). We observed similar effects in the same direction for % poverty, % unemployment, % < HS education, % homes built pre-1950 and Diversity Index (Beta coefficient = -0.043, -0.071, -0.044, -0.019, -1.409, respectively, with p < 0.001 in all these tests). Conversely, 1% increase in homeownership in census tracts would increase the distance by 0.022 km (p < 0.001). For income-related variables, a significant increase in income was observed with an increase in distance between a census tract and the closest TRI facility (1.15 ×10^-5^ for median HH income, p < 0.001).

**Table 3:** Linear regression of decay in distance to TRI facilities by exposure factors in Maryland (2010)

	**Univariate linear regression**		**Multivariate linear regression**	
**Sociodemographic measures**	**Beta coefficient**	**p-value**	**Beta coefficient**	**p-value**
**% Hispanic**	-0.014	0.01	0.028	<0.001
**% Non-white**	-0.011	<0.001	-	-
**% Poverty**	-0.043	<0.001	-	-
**% Unemployment**	-0.071	<0.001	-	-
**% < HS education**	-0.044	<0.001	-	-
**% Homeownership**	0.022	<0.001	0.006	<0.046
**% Homes built pre-1950**	-0.019	<0.001	-0.017	<0.001
**Diversity index**	-1.409	<0.001	-2.523	<0.001
**Median HH income**	1.15 × 10^-5^	<0.001	0.85 × 10^-5^	<0.001
**HPSA**	-0.717	<0.001	-	-

-: variables in the initial multivariate model before stepwise model selection (both direction) based on BIC and confounder selection.

In the multivariate regression model, after performing stepwise variable selection (Additional file 1: Table S1) and evaluating the confounding effects of the SOD factors (Additional file 2: Table S2), % non-white, % homes built pre-1950, Diversity index and median HH income were shown to be significantly associated with TRI facility distance from census tracts (p < 0.001) with % homeownership as a potential confounder. The direction of the effects remained the same except for % Hispanic. The direction of the association between distance to TRI facilities and % Hispanic changed from negative to positive. This is likely due to the adjustment for potential confounders (e.g., % unemployment, % less than HS education, and % homeownership).

Table 4 presents mean distribution of SOD measures in HPSA and non-HPSA tracts based on distance to TRI facilities from census tracts. There were 57 HPSA tracts and 202 non-HPSA tracts that host TRI facilities. There were 117 HPSA tracts and 496 non-HPSA tracts in areas where the distance to the nearest TRI facility from a census tract was from 1 km to 5 km. In general, % non-white was higher in HPSA tracts that hosted at least one TRI facility than in non-HPSA tracts that hosted at least one TRI facility. Additionally, % poverty, % unemployment, % less than HS education, % homeownership, and % homes built pre-1950 were higher in HPSA tracts hosting TRI facilities than in non-HPSA tracts hosting TRI facilities. All the means of SOD measures in HPSA and non-HPSA tracts were statistically different except for % Hispanic in both host tracts and tracts with nearest TRI facilities at a distance from 1 km to 5 km away.

**Table 4:** Mean distribution of sociodemographic measures by TRI facility buffer zones for 2010 Maryland HSPA tracts and non-HPSA tracts

**Sociodemographic measures**	**Host**	**1 km – 5 km buffer**
	**HPSA/Non-HPSA**	**HPSA/Non-HPSA**
**# Census tracts**	57/202	117/496
**% Hispanic**	8.3/6.6*	10.3/8.2*
**% Non-white**	55.8/33.4	79.8/41.9
**% Poverty**	18.8/8.8	16/6.5
**% Unemployment**	11.2/6.2	11.1/5.5
**% Less than HS education**	22.2/13.3	21/9.5
**% Homeownership**	53.4/68.6	47.9/73.8
**% Homes built pre-1950**	41.5/20.9	27.6/14.4
**Diversity index**	0.41/0.43*	0.37/0.45
**Median HH income**	47428/70202	48723/88798

*Statistically insignificant at the level of 0.05.

Overall, statistically significant higher levels of % non-white, % poverty, % unemployment, % less than HS education and % homes built pre-1950 were observed in HPSA tracts than in non-HPSA tracts, regardless of whether or not those areas hosted a TRI facility. Additionally, % homeownership was lower in HPSA tracts compared to non-HPSA tracts. For the Diversity Index, in areas within 1 km to 5 km to a TRI facility, non-HPSA tracts had a higher index than HPSA tracts. Median HH income was higher in HPSA tracts than non-HPSA tracts, regardless of whether or not the tract hosted a TRI facility.

## Discussion

Our results primarily indicate that people of color, low-income populations, and persons with less than HS education are located closer to TRI facilities than other groups or there are greater numbers of people of color and low-income persons in census tracts in areas that host TRI facilities. However, we did observe a lag effect of TRI facilities on the distribution of non-whites across different distance bands with higher percent non-white in the third distance band (>0.5 km and <1 km) compared to the host band (0 km) and the second distance band (>0 km and <0.5 km). For % poverty and % less than HS education, we did not observe this lag effect. Univariate regression results reveal statistically significant inverse relationships between distance to TRI facilities and % Hispanic, % non-white, % poverty, % unemployment, % less than HS education, % homes built pre-1950, and Diversity index (all statistically significant). Conversely, a positive relationship was observed between distance to TRI facilities and % homeownership and median HH income. In the multivariate models, results reveal statistically significant inverse relationships between distance to TRI facilities and % homes built pre-1950 and Diversity Index, and a positive relationship between distance and median HH income and % homeownership. Percent Hispanic changed from negative to positive which may be due to adjustment of other SOD factors in the multivariate model that could act as confounders.

The results of our study mirror results of previous research that demonstrated burden disparities in the distribution of TRI facilities and other LULUs based on race/ethnicity and class [6-18]. The results of this study are most similar to results of recent work in metropolitan Charleston [9] and St. Louis [16] where more TRI facilities were located in census tracts with higher non-white and low-income populations. Additionally, Fricker and Hengarter found a direct relationship between racial composition and presence of TRI facilities [7]. Both Ringquist and Neumann et al found TRI facilities were located in people of color neighborhoods [11,15]. Unlike Ringquist [15], we did not use zip codes but census tracts as the unit of analysis.

A benefit of including HPSA data is that we can compare HPSA census tracts to non-HPSA census tracts to assess relative differences in the mean distribution of SOD measures by TRI host and buffer zones. This approach is to understand if people of color and disadvantaged groups are both overburdened and medically underserved which is a problem when addressing environmental health disparities. For example, we observed that % poverty in HPSA census tracts (tracts with insufficient access to primary care) hosting a TRI facility was more than twice the % poverty in the equivalent non-HPSA census tracts (18.8% and 8.8%, respectively). Furthermore, we found that % < HS education population in HPSA census tracts hosting TRI facilities was almost twice that of non-HPSA census tracts (22.2% and 13.3%, respectively).

Aside from disparities in mean poverty between HPSA and non-HPSA TRI host census tracts, non-white populations had a higher percentage of persons hosting TRI facilities in HPSA versus non-HPSA tracts (non-white: 55.8%, 33.4%, respectively). Additionally, we observed disparities in % non-white, % poverty, % unemployment, and % less than HS education and also median HH income in the 1 km to 5 km buffer for HPSA vs non-HPSA tracts. The disparity in race, SES, and educational attainment in HPSA versus non-HPSA census tracts is problematic because a higher percentage of these populations live in areas hosting TRI facilities and they lack the salutogenic infrastructure required to act as a buffer against exposure to toxic emissions and other environmental stressors.

This study had several strengths with the first being that all data were from the same year (2010). In studies that use census data, this can only be said once a decade. In addition, this work can contribute to statewide planning as part of PlanMD [68] which is Maryland’s comprehensive plan for sustainable growth and development. While these analyses were specific to MD, the methods can be used as a template for other states trying to illustrate the same relationships among SOD composition, presence of pollution-emitting facilities, and health care infrastructure. Another notable strength of this study is that it provides insight into SOD measures other than race/ethnicity that may be a better indicator of spatial disparities in the distribution of TRI facilities within census tracts. Furthermore, our analysis of HPSA versus non-HPSA census tracts hosting TRI facilities can be used by the state to guide the allocation of resources to help reduce toxic releases in vulnerable communities.

After completion of this study, there is still a need for further analyses. We did not measure actual exposure in fenceline communities near each TRI facility or the various buffer zones but rather used distance as a proxy for exposure to TRI facilities. In addition, we did not include TRI emissions data or measure any health outcome data in the buffer zones to show differential health status in conjunction with the location of TRI facilities. However, it is worth noting that in terms of effect magnitude some findings we identified via multivariate linear regression were not significant, but they provide an overall trend in the population under study. In reference to HPSA status, additional research is needed to address the potential “double disparity” effect for non-white and economically disadvantaged communities who are living in HPSA tracts that were disproportionately burdened by TRI facilities and may have differential exposure to toxins reported and not reported by facilities under EPCRA.

In the future, we plan to incorporate Risk-Screening Environmental Indicators (RSEI) and National-Scale Air Toxics Assessment (NATA) data from the USEPA in order to conduct a more comprehensive analysis. Specifically, the RSEI database provides information regarding the amount of chemical releases, the fate and transport of the chemical, the route and extent of human exposure to the chemical, the number of people affected, and toxicity [69] which would allow us to better estimate exposure to TRI emissions. Using NATA data would allow us to estimate cancer risk so that we could determine whether there is a disparity in cancer risk in communities hosting TRI facilities. In addition we plan to further examine trends in facility siting and changes in emissions over time from 1990, 2000, and 2010.

One final consideration is whether the use of census tracts is the most appropriate population measure. While there is a wealth of information available at the census tract level, they are often not representative of true neighborhood boundaries. If we decide to represent a real neighborhood analysis in the future, we will have to move away from the use of zip code and census tract analyses in our research particularly for metropolitan statistical areas. We see potential in the approach taken by the Baltimore Neighborhood Indicators Project [70] to measure and track ‘actual’ neighborhood level social, environmental, and health data. We also believe that the use of planning districts or councilmatic districts may be a useful alternative to census tracts.

## Conclusion

This information may be useful to community-based organizations seeking to obtain information on the spatial distribution of TRI facilities and assistance from federal agencies such as the USEPA and the Agency for Toxic Substances and Disease Registry (ATSDR) to study the negative health impacts of these sites as part of a comprehensive community revitalization program. In addition, state agencies such as the MD Department of the Environment and the MD DHMH may be able to use the results of this study in its efforts to prioritize areas in vulnerable communities with a high concentration of TRI facilities and toxic releases and leverage state resources to clean up areas, improve public health, and enhance quality of life and community sustainability.

Additionally, this work has utility in providing metrics for how federal and state regulatory programs are meeting goals to reduce environmental injustice and environmental health disparities including cumulative impacts of environmental hazards in environmental justice communities. In addition, this work can contribute to statewide efforts to reduce health disparities and achieve health equity through implementation of the Affordable Care Act or state laws such as the MD Health Improvement and Health Disparities Reduction Act. With the right investment of ACA resources, hospitalizations, emergency room visits, and overall burden of disease related to exposure to toxins and other agents could be reduced in overburdened and underserved areas.

## Abbreviations

TRI: Toxic release inventory; SOD: Sociodemographic; USEPA: United States Environmental Protection Agency; HPSA: Health Professional Shortage Area; SES: Socioeconomic status; MDHMH: Maryland Department of Health and Mental Hygiene; EPHTN: Environmental Public Health Tracking Network; NATA: National-Scale Air Toxics Assessment; MD: Maryland; GIS: Geographic information systems; EPCRA: Emergency Planning and Community Right-to-Know Act; RSEI: Risk-Screening Environmental Indicators; HH: Household; HS: High school; HEZ: Health Enterprise Zone; MHIHDRA: Maryland Health Improvement and Health Disparities Reduction Act; POTWs: Publicly Operated Treatment Works; LULUs: Locally unwanted land uses; MPEMHD: Maryland Plan to Eliminate Minority Health Disparities; DHHS: Department of Health and Human Services; MSA: Metropolitan statistical area; FRS: Federal Registry System; ATSDR: Agency for Toxic Substances for Disease Registry; BIC: Bayesian Information Criterion.

## Competing interests

There are no financial or non-financial competing interests to disclose.

## Author’s contributions

RR drafted the manuscript. CJ designed and performed the statistical analysis and mapping. KB assisted with drafting, reviewing, and editing the manuscript. RM assisted with reviewing and editing the manuscript. HZ assisted with reviewing and editing the manuscript. CN assisted with reviewing and editing the manuscript. SW designed the study and assisted with drafting the manuscript. All authors read and approved the final manuscript.

## Supplementary Material

Additional file 1: Table S1 BIC Stepwise Model Selection on Both Directions. Click here for file

Additional file 2: Table S2 Confounder Selection (Each possible confounder was added sequentially). Click here for file
