# Supplementary material for: Being overburdened and medically underserved: assessment of this double disparity for populations in the state of Maryland
Source: Environ Health. 2014 Apr 4;13:26. doi: 10.1186/1476-069X-13-26 (PMC4021052; doi:10.1186/1476-069X-13-26)
Supplement: Additional file 1: Table S1 — BIC Stepwise Model Selection on Both Directions. [file 1476-069X-13-26-S1.docx]

**Additional file 1: Table S1** BIC Stepwise Model Selection on Both Directions

| Model Selection | Steps | Deviance | BIC | R^2^ |
| --- | --- | --- | --- | --- |
| Full model* |  |  | 2021.57 | 0.113 |
| - HPSA | 1 | 0.18 | 2014.37 | 0.114 |
| -% Unemployment | 2 | 2.41 | 2007.73 | 0.114 |
| -% Poverty | 3 | 7.52 | 2002.33 | 0.113 |
| -% Non-White | 4 | 7.64 | 1996.97 | 0.113 |
| -% Less than HS Education | 5 | 14.77 | 1993.34 | 0.111 |
| -% Homeownership+ | 6 | 16.44 | 1990.11 | 0.109 |

*: Full model includes: % Hispanic, % Non-White, %Poverty, %Unemployment, % Less than HS Education, % Homeownership, % Homes Built pre-1950, Diversity Index, Median HH Income, and HPSA.

The symbol ‘-‘means that the model removes the variable in that step. Within each step, the model also tried adding a variable, but as seen in the table, no variables were added during this stepwise selection process.

+: Final model includes: % Hispanic, % Homeownership, % Homes Built pre-1950, Diversity Index, and Median HH Income.
