# Supplementary material for: Being overburdened and medically underserved: assessment of this double disparity for populations in the state of Maryland
Source: Environ Health. 2014 Apr 4;13:26. doi: 10.1186/1476-069X-13-26 (PMC4021052; doi:10.1186/1476-069X-13-26)
Supplement: Additional file 2: Table S2 — Confounder Selection (Each possible confounder was added sequentially). [file 1476-069X-13-26-S2.docx]

**Additional file 2: Table S2** Confounder Selection (Each possible confounder was added sequentially)

| Diversity | % Homes Built pre-1950 | Median HH Income | % Hispanic | % Non-White | % Poverty | % Unemployment | % < HS education | % Homeownership | HPSA | R^2^ |
| --- | --- | --- | --- | --- | --- | --- | --- | --- | --- | --- |
| -2.734+ | -0.017+ | 1.11 × 10-5+ | 0.029+ |  |  |  |  |  |  | 0.108 |
| -2.582+ | -0.017+ | 9.8 × 10-6+ | 0.031+ | -0.0044* |  |  |  |  |  | 0.111 |
| -2.58+ | -0.017+ | 9.5 × 10-6+ | 0.031+ | -0.0043* | -0.002 |  |  |  |  | 0.110 |
| -2.578+ | -0.017+ | 9.2 × 10-6+ | 0.030+ | -0.0044* | -0.002 | 9.10 × 10-5 |  |  |  | 0.110 |
| -2.577+ | -0.017+ | 9.1 × 10-6+ | 0.030+ | -0.0044* | -0.002 | 7.70 × 10-5 | 7.00 × 10-5 |  |  | 0.110 |
| -2.432+ | -0.017+ | 7.5 × 10-6** | 0.030+ | -0.0036 | 0.002 | 6.80 × 10-5 | 1.00 × 10-4 | 0.0059 |  | 0.111 |
| -2.514+ | -0.017+ | 7.3 × 10-6** | 0.030+ | -0.003 | 0.002 | 6.80 × 10-5 | 0.00011 | 0.0058 | -0.13 | 0.110 |

+: p-Value <0.001; **: p Value <0.01; * p Value <0.05.

Since the inclusion of % homeownership changed the coefficient of median HH income by >10%,%homeownership was treated as a potential confounder and included in our final model.

Note: *:- Statistically insignificant at the level of 0.05.
